# Hypoimmunogenic CD19 CAR-NK cells derived from embryonic stem cells suppress the progression of human B-cell malignancies in xenograft animals

**DOI:** 10.3389/fimmu.2024.1504459

**Published:** 2024-11-27

**Authors:** Qi Zhang, Chengxiang Xia, Qitong Weng, Leqiang Zhang, Yao Wang, Yanhong Liu, Xiujuan Zheng, Yunqing Lin, Yi Chen, Yiyuan Shen, Hanmeng Qi, Lijuan Liu, Yanping Zhu, Min Zhang, Dehao Huang, Fangxiao Hu, Mengyun Zhang, Hui Zeng, Jinyong Wang, Tongjie Wang

**Affiliations:** ^1^ State Key Laboratory of Stem Cell and Reproductive Biology, Institute of Zoology, Chinese Academy of Sciences, Beijing, China; ^2^ Beijing Institute for Stem Cell and Regenerative Medicine, Beijing, China; ^3^ Department of Hematology, The First Affiliated Hospital of Jinan University, Guangzhou, China

**Keywords:** embryonic stem cells, CD19 CAR-NK cells, hypoimmunogenicity, cytotoxicity, B-cell malignancy

## Abstract

**Background:**

Chimeric antigen receptor (CAR) engineered natural killer (NK) cells exhibit advantages such as MHC-independent recognition and strong anti-tumor functions. However, allogeneic CAR-NK cells derived from human tissues are heterogeneous and susceptible to clearance by hosts.

**Methods:**

We generated a B2M knockout, HLA-E and CD19 CAR ectopic expressing embryonic stem cell (ESC) line, which differentiated normally and gave rise to homogeneous CD19 CAR-NK (CD19 CAR-UiNK) cells using an organoid aggregate induction method. The CD19 CAR-UiNK were co-cultured with T cells or NK cells derived from peripheral blood mononuclear cells (PBMC) with the mismatched HLA to evaluate the immunogenicity of CD19 CAR-UiNK cells. We further assessed the therapeutic effects of CD19 CAR-UiNK cells on CD19^+^ tumor cells through *in vitro* cytotoxicity assays and *in vivo* animal models.

**Results:**

The CD19 CAR-UiNK cells exhibited typical expression patterns of activating and inhibitory receptors, and crucial effector molecules of NK cells, similar to those of unmodified NK cells. In co-culture assays, the CD19 CAR-UiNK cells evaded allogeneic T cell response and suppressed allogeneic NK cell response. Functionally, the CD19 CAR-UiNK cells robustly secreted IFN-γ and TNF-α, and upregulated CD107a upon stimulation with Nalm-6 tumor cells. The CD19 CAR-UiNK cells effectively eliminated CD19^+^ tumor cells *in vitro*, including B-cell cancer cell lines and primary tumor cells from human B-cell leukemia and lymphoma. Further, the CD19 CAR-UiNK cells exhibited strong anti-tumor activity in xenograft animals.

**Conclusion:**

We offer a strategy for deriving homogeneous and hypoimmunogenic CD19 CAR-iNK cells with robust anti-tumor effects from ESCs. Our study has significant implications for developing hypoimmunogenic CD19 CAR-NK cell therapy using human ESC as an unlimited cell source.

## 1 Introduction

CAR-NK cells have emerged as a promising option for immunotherapy due to their minimal toxicities and MHC-independent recognition (1). Currently, NK cells used in clinical trials are primarily from the NK-92 cell line, peripheral blood, or umbilical cord blood (2). However, these NK cells face challenges, including functional heterogeneities, low efficiencies, and high gene engineering or editing costs.

Human pluripotent stem cells (hPSCs) are promising cell sources for the production of standardized, off-the-shelf NK cells, offering advantages such as unlimited cell sources and the ability to perform multiple genetic modifications (3). By harnessing these advantages, hPSCs can be engineered to generate NK cells with enhanced anti-tumor capabilities (4, 5). For example, by stably integrating a high-affinity, non-cleavable version of CD16a into human induced pluripotent stem cells (hiPSCs), the NK cells can be derived from this modified hiPSCs and show improved ADCC for clinical applications (6). Moreover, CARs recognizing tumor antigens (CD19 and mesothelin) have been effectively expressed in hPSC-NK cells (7, 8).

Allogeneic CAR-NK cells are associated with less toxic effects compared to CAR-T cells (9, 10). However, recipients’ immune rejection of allogeneic CAR-NK cells reduces their therapeutic effects and limits their use. Highly polymorphic human leukocyte antigens (HLA) help the host differentiate self-cells from non-self-cells and play a central role in T cell-mediated graft rejection. Depleting HLA molecules on allogeneic cells before therapy is considered a major strategy to evade host T cell recognition and attack. β2-microglobulin (B2M), which encodes a common protein subunit of HLA-I complex, is required for the formation of all polymorphic HLA class I heterodimers (11–16). Class-II major histocompatibility complex transactivator (CIITA) is a master regulator for determining the HLA class II expression (17, 18). Several recent studies have explored the generation of hypoimmunogenic hPSC-derived T cells, cardiomyocytes(Deuse et al., 2019; Mattapally et al., 2018), and endothelial cells by knocking out B2M and CIITA, which prevented CD8^+^ T and CD4^+^ T cell-mediated allo-rejections (19–21). However, the loss of HLA class I triggers NK cells’ “missing self” killing response by NK cells (22, 23). Nonpolymorphic HLA class Ib molecules HLA-E and HLA-G, which bind the NK cell inhibitory receptor CD94/NKG2A and ILT2 respectively, can suppress NK cell activation, both of which are highly expressed on extravillous trophoblasts and are involved in placental NK tolerance (24–27). Accordingly, some studies introduced HLA-E or HLA-G into hPSCs to derive functional cells avoiding host NK cell activation and rejection, including T cells, CD45^+^ hematopoietic cells, cardiomyocytes, and vascular smooth muscle cells (11, 19, 28, 29). Moreover, HLA class Ia molecules are the cognate ligands for inhibitory killer immunoglobulin-like receptors of NK cells (30, 31). Retaining one allele of HLA-C gene or HLA-A gene in hPSCs while depleting other HLA class Ia molecules avoided NK cell-mediated rejection of hPSC-derived blood cells or endothelial cells (32, 33). Introducing the HLA-A*11:01 allele into B2M KO iPSCs (A11-B2M KO-iPSC) reduced NK cell rejection against A11-B2M KO-iPSC derived endothelial cells (34). Recently, an alternative strategy was explored to evade NK cell rejection by overexpressing the immune checkpoint inhibitor CD47, which binds the signal regulatory protein α (SIRPα) on allogenic donor cells (21, 35–38). However, other groups have found only modest or no impacts of CD47 blockade on NK cell activity or toxicity (19, 39). These conflicting reports may be due to differences in cell types, cell modifications, and experimental systems. Currently, effective methods have been developed for inducing hPSCs into NK cells (40, 41). Nonetheless, further exploration is needed to induce universal NK cells from hPSCs for clinical translation.

Here, we established a human ESC cell line with B2M knockout and ectopic expression of HLA-E and CD19 CAR (CD19 CAR-UESC). The CD19 CAR-UESC efficiently generated CD19 CAR-UiNK cells using an organoid aggregate induction method we established (40). Deleting of HLA-I molecules and the lack of endogenous HLA-II molecule expressions allowed the CD19 CAR-UiNK cells to escape immune rejection by allogeneic T cells. Additionally, the enforced expression of HLA-E on CD19 CAR-UiNK cells inhibited the attack by allogeneic NK cells. The CD19 CAR-UiNK cells effectively eliminated CD19^+^ tumor cells *in vitro*, including B-cell cancer cell lines and primary cells from human B-cell leukemia and lymphoma. Notably, the CD19 CAR-UiNK cells suppressed the tumor progression and prolonged survival in Nalm-6 tumor xenograft animals. Our study provides a strategy for deriving hypoimmunogenic CD19 CAR-NK cells from ESCs for potentially treating human B-cell malignancies.

## 2 Materials and methods

### 2.1 Cell culture

The human ESC line was provided by the National Stem Cell Resource Center, Institute of Zoology, Chinese Academy of Sciences. ESC line was maintained in Essential 8 medium (Gibco) on vitronectin (Gibco) coated plates. The OP9 cell line was purchased from ATCC and cultured with α-MEM (Gibco) with 20% fetal bovine serum (FBS) (Ausbian). Primary human leukemia and lymphoma cells were isolated from the bone marrow of three patients, with the donor’s informed consent. Luciferase-expressing Nalm-6 cells, kindly provided by Professor Min Wang (Leukemia Center, Institute of Hematology and Blood Diseases Hospital, Chinese Academy of Medical Sciences, Tianjin, China), were cultured in RPMI 1640 medium (Gibco) supplemented with 10% FBS. Raji cells (Pricella Biotechnology) were cultured in RPMI 1640 medium (Gibco) supplemented with 10% FBS.

### 2.2 B2M knockout in ESCs

Two gRNA sequences (gRNA1: gctgtgctcgcgctactctc, gRNA2: ggcttgacttcaatctcgat) targeting exon 1 and intron 1 of B2M were designed using a web-based guide-RNA designer (http://www.rgenome.net/) and cloned into the Cas12i^HiFi^ expression vector (a gift from Dr. Wei Li of the Institute of Zoology, Chinese Academy of Sciences) (42). To generate B2M KO-ESC, the two Cas12i^HiFi^-sgRNA expression vectors were co-transfected into ESCs by electroporation. After editing, the ESCs were incubated with FITC anti-human HLA-ABC antibody (clone: W6/32, Biolegend, 311404) on ice for 15-30 min. HLA-ABC negative ESCs were sorted by Sony MA900 Sorter.

### 2.3 HLA-E and CD19 CAR transduction in B2M KO-ESC

The fragment encoding a single chain trimer (SCT) of HLA-E consisting of, in the following order (from N- to C-terminus), the signal peptide of human β2m, a nonameric peptide VMAPRTLFL, (G4S)_3_ flexible linker, mature human β2m, (G4S)_3_ flexible linker, and HLA-E*01:01 heavy chain was cloned and assembled to the PiggyBac vector (PB530A-2, SBI). The single-chain Fv (scFv) specific for CD19 (43) was used for CAR construction (CD19 scFV-CD8α hinge-CD8α TM-CD3ζ). The CAR construct was cloned into the PiggyBac expression vector (PB530A-2, SBI) to generate the PB-EF1α-CD19 CAR vector. Then, the HLA-E expression PiggyBac vector, PB-EF1α-CD19 CAR vector, and the transposase expression vector were electroporated into B2M KO-ESC together using Electroporator EX+ (Celetrix, 11-0106). Following electroporation, ESCs were stained with anti-human HLA-E APC (Biolegend, 342606) and Mouse anti-mouse FMC63 Alexa Fluor 647 (BioSwan, 300402), and sorted by BD FACSAria™ Fusion for two rounds to establish the stable CD19 CAR and HLA-E-expressing B2M KO-ESC (CD19 CAR-UESC).

### 2.4 Generation of iNK, B2M KO-iNK, or CD19 CAR-UiNK cells

The derivation of iNK cells from pluripotent stem cells has been previously described (40). Briefly, B2M KO-ESC, CD19 CAR-UESC, and ESC were subjected to a two-day monolayer induction to acquire highly purified lateral plate mesoderm cells. Subsequently, 2×10^4^ lateral plate mesoderm cells and 5×10^5^ OP9 feeder cells were assembled into organoid aggregates and seeded onto transwell inserts, establishing an air-liquid interface for hematopoietic differentiation. Mature iNK, B2M KO-iNK, or CD19 CAR-UiNK cells were obtained after 25 days of induction.

### 2.5 RNA-seq and data analysis

The CD19 CAR-UiNK cells and iNK cells (CD45^+^CD3^-^CD56^+^) sorted on Day 27 were used for 10× scRNA-seq. Droplet-based scRNA-seq datasets were produced using a Chromium system (10× Genomics, PN120263) following the manufacturer’s instructions. Droplet-based scRNA-seq datasets were aligned to reference genome GRCh19 and quantified using the Cell Ranger software package (version 7.0) and subjected to Seurat (version 4.3.0) for further analysis. Projection of CD19 CAR-UiNK onto iNK was performed using the Seurat package. Before integrating data, we performed subsequent quality control (QC). To pass QC, cells were required to have less than 10% of aligned reads mapping to mitochondrial genes and less than 5% of aligned reads mapping to hemoglobin genes. All datasets were integrated using Seurat’s integration function. The standard workflow for UMAP dimensionality reduction was performed using the top 30 PCs. Violin plots for gene expression were plotted using the VlnPlot function of Seurat and the ggplot2 package. All the raw data (fastq files) were uploaded to the Genome Sequence Archive public database (HRA001609, HRA007737).

### 2.6 Flow cytometry

Cells were blocked by Human TruStain FcX™ (Biolegend, 422302) antibody, and then stained with related antibodies. The following antibodies were used: anti-human CD45 (Biolegend, HI30), anti-human CD3 (Biolegend, HIT3a), anti-human CD16 (Biolegend, 3G8), anti-human CD56 (Biolegend, HCD56), anti-human 2B4 (Biolegend, C1.7), anti-human DNAM-1(Biolegend, 11A8), anti-human NKp30 (Biolegend, P30-15), anti-human NKp46 (Biolegend, 29A1.4), anti-human NKG2D (Biolegend, 1D11), anti-human NKG2A (Biolegend, S19004C), anti-human CD96 (Biolegend, NK92.39), anti-human CD94 (BD Biosciences, HP-3D9), anti-human CD69 (Biolegend, FN50), anti-human TRAIL (Biolegend, RIK-2), anti-human GzmB (Biolegend, QA18A28), anti-human Perforin (Biolegend, dG9), Mouse anti-mouse FMC63 (Bioswan), anti-human HLA-A, B, C (Biolegend, W6/32), and anti-human HLA-E (Biolegend, 3D12). The cells were resuspended in the DAPI (Sigma-Aldrich) solution and were analyzed with BD LSR Fortessa X-20 cytometer (BD Biosciences). Flow cytometry data were analyzed by the FlowJo software (Three Star, Ashland OR).

### 2.7 T-cell proliferation assay

HLA-typed allogeneic PBMCs were purchased from SCHBIO Inc (Shanghai, China). Upon thawing, PBMCs were labeled with Cell Proliferation Dye eFluor™ 670 (Invitrogen) following the manufacturer’s instructions. Three groups of iNK, B2M KO-iNK, and CD19 CAR-UiNK cells were irradiated (with 30 Gy) to stop proliferation. Effector eFluor™ 670-stained PBMCs were mixed with the targets (iNK, B2M KO-iNK, and CD19 CAR-UiNK) at a ratio of 10:1 in 200 μL RPMI-1640 supplemented with glutamine, 10% heat-inactivated human FBS, and 25 ng/mL IFN-γ. PBMCs cultured without target cells used as a negative control were called MOCK. On day 7, cells were stained with anti-human CD3-FITC (Biolegend, 300406), anti-human CD4-PE/CY7 (Biolegend, 357410), and anti-human CD8-BV605 (Biolegend, 344742) antibodies. Subsequently, cells were stained with DAPI. The percentage of proliferative CD3^+^ CD8^+^ T cells and CD3^+^ CD4^+^ T cells that became negative for eFluor™ 670 labeling was analyzed on a BD LSRFortessa.

### 2.8 T cell cytotoxicity assay

Primed eFluor™ 670-negative CD8^+^ T cells that reacted to allogeneic iNK cells were sorted and co-cultured in 2 μg/mL PHA, 10 ng/mL IL-7, 5 ng/mL IL-15, and 200 U/mL IL-2 with irradiated PBMC feeders. Activated CD8^+^ T cells were used after an expansion period of 10 to 15 days. For T cell cytotoxicity assays, iNK, B2M KO-iNK, and CD19 CAR-UiNK cells were labeled with Cell Proliferation Dye eFluor™ 670 (Invitrogen) following the manufacturer’s instructions and were mixed with activated CD8^+^ T cells at the effector target ratios of 20:1, 10:1, 5:1, 2:1, and 1:1 in 200 µl RPMI-1640 supplemented with glutamine, 10% heat-inactivated human FBS. After 22 hours, cells were stained with DAPI and analyzed by flow cytometry. Then, the lysis of iNK, B2M KO-iNK, and CD19 CAR-UiNK cells by CD8^+^ T cells was determined by measuring the percentage of DAPI^+^ cells within the eFluor™ 670^+^ singlets.

### 2.9 NK cell cytotoxicity assay

PBMCs were purchased from SCHBIO Inc (Shanghai, China). Upon thawing, PBMCs were sequentially incubated with Biotin anti-human CD3 antibody (Biolegend, 300304) and anti-biotin microbeads (Miltenyi Biotec). Then, CD3^-^ PBMCs were purified after magnetic separation. Purified CD3^-^ PBMCs were stimulated with K562-mIL-21 cells (Hangzhou Zhongying Biomedical Technology Co., Ltd) and recombinant human IL-2 (Miltenyi Biotec, 200 U/mL) in KBM581 Serum-free Medium (Corning) for NK cell activation and expansion. After 11-14 days, iNK, B2M KO-iNK, and CD19 CAR-UiNK cells were labeled with Cell Proliferation Dye eFluor™ 670 (Invitrogen). Labeled cells (targets, T) were mixed with activated PBMC-NK cells (effectors, E) at the E: T ratios of 3:1, 1:1, and 0.3:1 in 200 µL RPMI-1640 supplemented with glutamine, 10% heat-inactivated human FBS. After 20 hours, cells were stained with DAPI and analyzed by flow cytometry. Then, the lysis of iNK, B2M KO-iNK, and CD19 CAR-UiNK cells by activated PBMC-NK was determined by measuring the percentage of DAPI^+^ cells within the eFluor™ 670 singlets.

### 2.10 CD107a degranulation assay, IFN-γ and TNF-α staining

iNK or CD19 CAR-UiNK cells (effectors, E) were incubated with or without Nalm-6 tumor cells (targets, T) at the E: T ratio of 0.5:1 for 4 hours. After incubation, cells were stained with CD107a (Biolegend, H4A3), CD45 (Biolegend, HI30), CD3 (Biolegend, HIT3a), and CD56 (Biolegend, HCD56). For IFN-γ and TNF-α staining, iNK or CD19 CAR-UiNK cells were incubated with or without Nalm-6 tumor cells (E: T=0.5:1) for 2 hours, followed by adding BFA/Monensin (MULTISCIENCES) for additional 2-hour incubation. After incubation, cells were stained with CD45 (Biolegend, HI30), CD3 (Biolegend, HIT3a), and CD56 (Biolegend, HCD56). FIX & PERM Kit (MULTISCIENCES) was used for fixation and permeabilization, followed by intracellular staining of IFN-γ (Biolegend, 4S.B3) or TNF-α (Biolegend, MAb11). The cells were analyzed with BD LSRFortessa X-20 cytometer (BD Biosciences). Flow cytometry data were analyzed by the FlowJo software (Three Star, Ashland OR).

### 2.11 Cytotoxicity assay

CD19 CAR-UiNK cells or iNK cells (effectors, E) were co-cultured with Nalm-6, Raji, or primary human B lymphoma or leukemia cells (targets, T) labeled with carboxyfluorescein diacetate succinimidyl ester (CFSE; Beijing BioRab Technology Co. Ltd.) in 96-well plates at respective E: T ratios (0.2:1, 0.4:1, 0.8:1, 1.6:1, or 5:1). The tumor cell lysis was analyzed at indicated times (4 hours for Nalm-6, 6 hours for Raji, and 12 hours for primary human B lymphoma and leukemia cells). In serial killing assay, CD19 CAR-UiNK cells or iNK cells were co-cultured with CFSE-labeled Nalm-6 tumor cells for 12 hours (Round 1) at E: T = 1:1. Fresh eFluor450-labeled Nalm-6 tumor cells were added into all wells co-cultured with the remaining effector cells for another 12 hours (Round 2) at the same E: T ratio. This process was repeated for a third round by adding eFluor670-labeled Nalm-6 tumor cells (Round 3). Target cell death was assessed with a flow cytometer (BD LSRFortessa X-20 cytometer, BD Biosciences) by the percentage of DAPI^+^ or 7-AAD^+^ in the labeled population. Flow cytometry data were analyzed by the FlowJo software (Three Star, Ashland OR).

### 2.12 Construction of the xenograft models and treatment with iNK/CD19 CAR-UiNK cells

NCG mice (NOD/ShiLtJGpt-*Prkdc* ^em26Cd52^ *Il2rg* ^em26Cd22^/Gpt, GemPharmatech Co., Ltd.) were intravenously injected with the luciferase-expressing Nalm-6 (Nalm-6-luci^+^) cells (2 × 10^5^ cells/mouse) on Day -1 to construct the B-ALL xenograft animal models. Bioluminescence imaging (IVIS Spectrum PerkinElmer) was performed on these models to quantify the tumor burden, and the models with similar total flux(p/s) were randomly divided into three groups (Tumor + PBS, Tumor + iNK, and Tumor + CD19 CAR-UiNK) on Day 0. These models were irradiated (2.25 Gy, Rad Source RS2000). Then, the mice were intravenously injected with PBS (200 μL), iNK cells (1–1.5 × 10^7^ cells/mouse), or CD19 CAR-UiNK cells (1-1.5 × 10^7^ cells/mouse) on Day 0 and Day 7. BLI was performed every week to trace the tumor cells. Mice suffering from heavy tumor burden were euthanized for ethical considerations.

### 2.13 Statistics

Data analyses were performed using GraphPad Prism. All data are represented as means ± SD, and the specific number (n) for each dataset is detailed in the figure legends. Two-tailed Student’s *t*-test, one-way ANOVA, two-way ANOVA, and log-rank test were used by SPSS software to calculate statistical significance. The results are notated as follows: NS, not significant; *P < 0.05; **P < 0.01; ***P < 0.001.

## 3 Results

### 3.1 Generation of CD19 CAR-UESC and induction of CD19 CAR-UiNK cells from genetically modified ESCs

**Figure 1:**
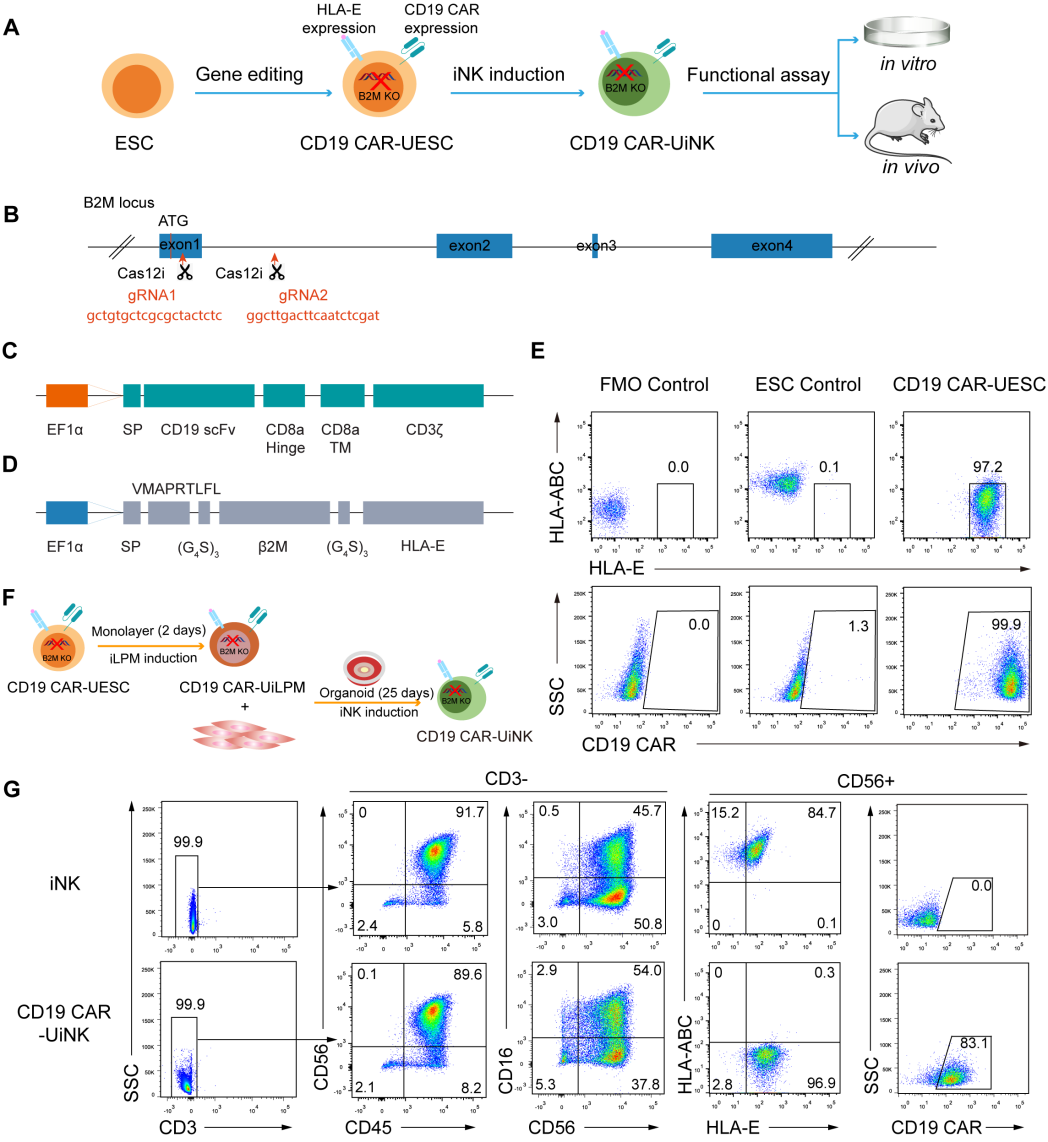
Generation of CD19 CAR-UESC and induction of CD19 CAR-UiNK. **(A)** Schematic diagram of CD19 CAR-UiNK generation and functional assay. **(B)** Targeting strategy for the knockout of *B2M* gene in human ESCs. The exons of the *B2M* gene are shown as blue boxes. Two gRNA sequences, gRNA1 and gRNA2, are marked in red. **(C)** Schematic representation of the transposon vector encoding the CD19 CAR. **(D)** Schematic representation of the transposon vector encoding the HLA-E single chain trimer. **(E)** Representative flow cytometry plots showing the expression of HLA-ABC, HLA-E, and CD19 CAR in ESC and CD19 CAR-UESC. **(F)** Schematic diagram of CD19 CAR-UiNK cell induction. **(G)** Representative flow cytometry plots of CD19 CAR-UiNK cells (CD45^+^CD3^–^CD56^+^CD16^+/–^HLA-ABC^–^HLA-E^+^CD19 CAR^+^) on Day 27.

To avoid immune rejection by host T and NK cells, we knocked out B2M and introduced both HLA-E and CD19 CAR into ESCs (CD19 CAR-UESC) to obtain universal CD19 CAR-iNK (CD19 CAR-UiNK) cells (**Figure 1A**). In our study, the NK cells derived from ESCs rarely express the HLA-II molecules (**Supplementary Figure 1A**), thus obviating the need for HLA-II molecule knockout. First, two distinct gRNAs were designed to target sequences within the first exon and first intron of the *B2M* gene (**Figure 1B**). We electroporated the vectors encoding these guides and Cas12i into the ESCs (42). After two rounds of sorting using an antibody that recognizes HLA-ABC, we obtained the *B2M* knockout ESCs (B2M KO-ESC). Subsequently, the CD19 CAR and HLA-E expressing cassette were integrated into ESCs using the PiggyBac transposon system. We designed an anti-CD19 CAR construct that consisted of a single-chain variable fragment (scFv), a CD8α hinge transmembrane region, and a CD3ζ activation domain (43, 44) (**Figure 1C**). HLA-E coding sequence was designed based on the previous research of Crew MD et al (45). It consists of a β2m signal peptide, an HLA-E binding peptide VMAPRTLFL (HLA-G signal sequence derived peptide), a flexible linker (G4S)_3_, mature human β2m, a flexible linker (G_4_S)_3_, and the HLA-E*01:01 heavy chain (**Figure 1D**). Electroporated ESCs were subjected to another two rounds of sorting using the antibodies that recognize HLA-E and CD19 CAR antigen. We achieved over 97% expression of HLA-E and over 99% expression of the CD19 CAR in these genetically modified ESCs (**Figure 1E**). Finally, we successfully established the CD19 CAR-UESC.

Previously, we developed an organoid aggregate method for generating human NK cells from pluripotent stem cells (PSCs) (40). Here, we used this method to generate CD19 CAR-UiNK cells from CD19 CAR-UESC. The unmodified ESCs were used as controls to monitor any disruptions in differentiation caused by genetic modification. Briefly, the lateral plate mesoderm (iLPM) cells were efficiently produced from CD19 CAR-UESC via monolayer induction for 48 hours. On day 2, the iLPM cells were mixed with OP9 feeder cells to prepare organoid aggregates. The organoids were plated on the transwell inserts and placed in 6-well plates for 25-day NK cell induction (**Figure 1F**). More than 90% of cells exhibited mature NK cell phenotypes (CD45^+^CD3^-^CD56^+^CD16^+/-^) on day 27. Flow cytometry analysis revealed that the CD19 CAR-UiNK cells barely express HLA-ABC. Over 98% of cells exhibited HLA-E, and over 83% expressed CD19 CAR (**Figure 1G**). The proliferation rate of the CD19 CAR-UiNK cells was similar to that of iNK cells *in vitro* (**Supplementary Figure 1B**). We further confirmed the absence of MHC-Class I and Class II in CD19 CAR-UESC and CD19 CAR-UiNK cells by flow cytometry analysis at baseline or following inflammatory stimulation (**Supplementary Figures 1C, D**). Thus, the CD19 CAR-UiNK cells are efficiently generated from CD19 CAR-UESC based on the organoid induction method we established.

### 3.2 Characterization of CD19 CAR−UiNK cells

**Figure 2:**
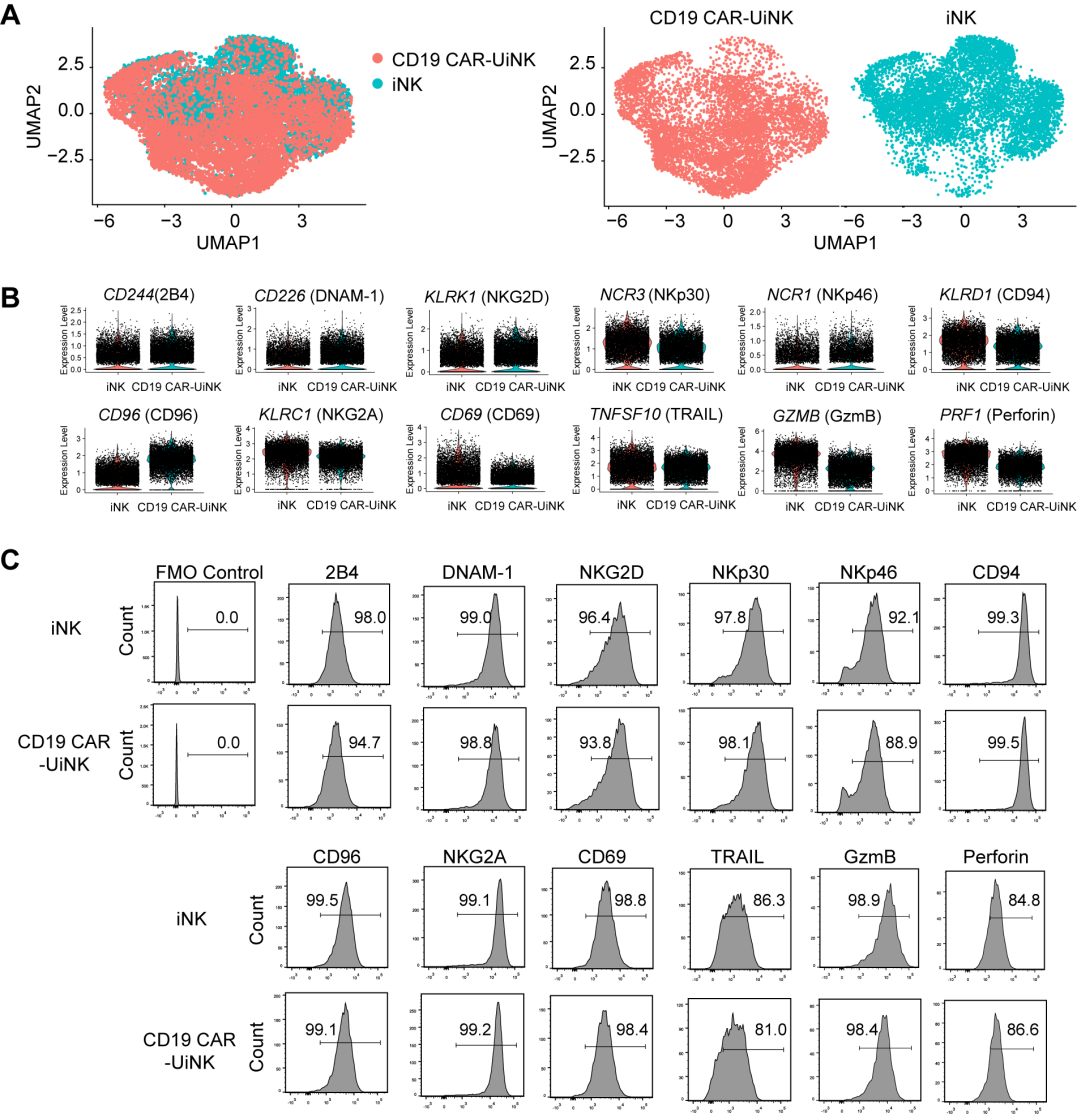
Molecular features of ESC-derived iNK and CD19 CAR-UiNK cells. **(A)** Projection of iNK and CD19 CAR-UiNK cells (left). UMAP visualization of iNK and CD19 CAR-UiNK cells respectively (right). **(B)** Violin plots showing the expression profiles of indicated NK cell-surface receptors and effectors (*CD244, CD226, KLRK1, NCR3, NCR1, KLRD1, CD96, KLRC1, CD69, TNFSF10, GZMB*, and *PRF1*) in iNK and CD19 CAR-UiNK cells. One point represents one cell. **(C)** Flow cytometry analysis of the expression levels of NK cell receptors and effectors (2B4, DNAM-1, NKG2D, NKp30, NKp46, CD94, CD96, NKG2A, CD69, TARIL, GzmB, and Perforin).

To elucidate the molecular characteristics of CD19 CAR-UiNK cells generated by CD19 CAR-UESC, we performed 10× single-cell RNA sequencing (scRNA-seq) of CD19 CAR-UiNK cells. The transcriptome of CD19 CAR-UiNK cells and iNK cells showed similar patterns, supported by scRNA-seq data showing a strong projection of CD19 CAR-UiNK cells onto iNK cells (**Figure 2A**). Both iNK and CD19 CAR-UiNK cells showed the expression of classical NK receptor genes, including activating receptor genes (*CD244*, *CD226*, *KLRK1*, *NCR3*, and *NCR1*) and inhibitory receptor genes (*CD96* and *KLRC1/KLRD1*) (46) (**Figure 2B**). The CD19 CAR-UiNK cells also exhibited high expression of crucial effector molecules, including apoptosis-related ligand (*TNFSF10*), cytotoxic granules (*GZMB* and *PRF1*), and activating molecule (*CD69*) (47–49) (**Figure 2B**). The flow cytometry results confirmed the expression of these molecules at protein level (**Figure 2C**). We have analyzed the pluripotency-associated genes of the iNK and CD19 CAR-UiNK cells using the RNA-seq data. The results showed no expression of pluripotency markers in the iNK cells and CD19 CAR-UiNK cells (**Supplementary Figures 2A, B**). Moreover, we confirmed that iNK cells do not express SSEA-4, one of the pluripotency markers, by flow cytometry analysis (**Supplementary Figure 2C**). In summary, the CD19 CAR-UiNK cells express typical NK cell molecules similar to iNK cells.

### 3.3 CD19 CAR-UiNK cells evade the allogeneic T cell response and suppress the allogeneic NK cell attack

**Figure 3:**
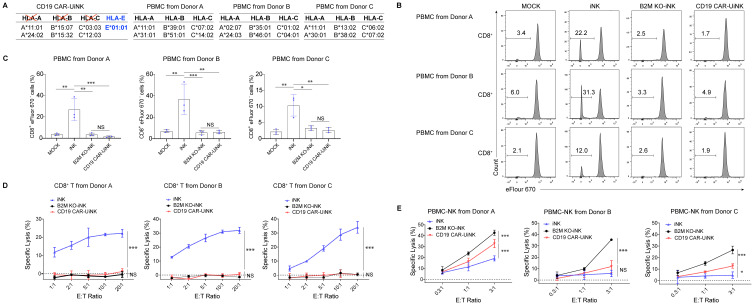
CD19 CAR-UiNK cells escape the response of allogeneic CD8^+^ T cells and NK cells. **(A)** HLA genotyping table for CD19 CAR-UiNK cells and donor PBMCs. (A, B, C, and E alleles). **(B)** Representative flow plots showing the percentage of proliferating CD8^+^ T cells. PBMC from three donors **(A–C)** were labeled with Cell Proliferation Dye eFluor 670 and co-cultured with irradiated iNK cells (iNK, B2M KO-iNK, or CD19 CAR-UiNK) for 7 days. PBMC cultured alone was defined as the MOCK group. **(C)** Statistical analysis of proliferating CD8^+^ T cell (n=3 donors) populations. Data are represented as means ± SD (n=3). NS, not significant, *P < 0.05, **P < 0.01, and ***P < 0.001 (one-way ANOVA). **(D)** HLA-reactive T cell cytotoxicity against iNK cells at the indicated effector: target (E: T) ratios. iNK, B2M KO-iNK, and CD19 CAR-UiNK cells (targets, T) were co-incubated with HLA-reactive T cells (effectors, E) for 22 hours. Specific lysis was calculated using the formula: (percentage of specific death – percentage of spontaneous death) × 100. Data are represented as means ± SD (n=3). NS, not significant, and ***P < 0.001 (Two-way ANOVA). **(E)** PBMC-NK cytotoxicity against iNK cells at the indicated effector: target (E: T) ratios. iNK, B2M KO-iNK, and CD19 CAR-UiNK cells (targets, T) were co-cultured with activated PBMC-NK cells (effectors, E) derived from Donor A, B, and C for 20 hours. Specific lysis was calculated using the formula: (percentage of specific death – percentage of spontaneous death) × 100. Data are represented as means ± SD (n=3). NS, not significant, *P < 0.05, and ***P < 0.001 (Two-way ANOVA).

Foreign HLA-I molecules can initiate an allogeneic T-cell response, resulting in graft rejection. To evaluate the immunogenicity of CD19 CAR-UiNK cells to allogeneic T cells, peripheral blood mononuclear cells (PBMC) from three donors with the mismatched HLA (**Figure 3A**; **Supplementary Figure 3A**) were labeled with Cell Proliferation Dye eFluor 670 and co-cultured with iNK, B2M KO-iNK, or CD19 CAR-UiNK cells. After seven days, the proliferation of the CD8^+^ T cells and CD4^+^ T cells was assessed by flow cytometry as the frequency of eFlour670^-^ cells. The data showed that co-culture with unmodified iNK cells activated alloreactive CD8^+^ T cells and induced their proliferation, as indicated by the increased percentage of eFlour670^-^ cells, whereas B2M KO-iNK and CD19 CAR-UiNK cells did not (**Figures 3B, C**). Due to the lack of HLA-II expression in ESC-derived induced NK cells, all of the iNK, B2M KO-iNK, and CD19 CAR-UiNK inhibited CD4^+^ T cell activation and proliferation (**Supplementary Figures 3B, C**). Further, the results of T cell cytotoxicity assays with HLA-I reactive CD8^+^ T cells indicated that iNK cells were killed by the alloreactive CD8^+^ T cells, but B2M KO-iNK and CD19 CAR-UiNK cells efficiently escaped the killing (**Figure 3D**). In short, CD19 CAR-UiNK cells can evade immune recognition.

Donor cells without HLA class I surface expression are vulnerable to NK-cell-mediated lysis through missing-self response. Contrary to mature NK cells fratricide after KO of B2M gene, the B2M KO-iNK cells were differentiated from PSC and proliferated normally *in vitro* (**Supplementary Figure 4**), indicating that the B2M-KO iNK cells didn’t suffer from the fratricide caused by downregulation of MHC class I. To evaluate whether CD19 CAR-UiNK cells with the forced HLA-E expression can inhibit allogeneic NK cell response, we incubated allogeneic PBMC-NK cells from healthy donors with iNK, B2M KO-iNK, or CD19 CAR-UiNK cells. The data showed that PBMC-NK cell-mediated cytotoxicity was reduced when co-incubated with CD19 CAR-UiNK cells (**Figure 3E**). Thus, CD19 CAR-UiNK cells effectively suppress allogeneic NK cell killing.

### 3.4 CD19 CAR-UiNK cells exhibited elevated IFN-γ, TNF-α, and CD107a expression upon stimulation with CD19^+^ tumor cells

**Figure 4:**
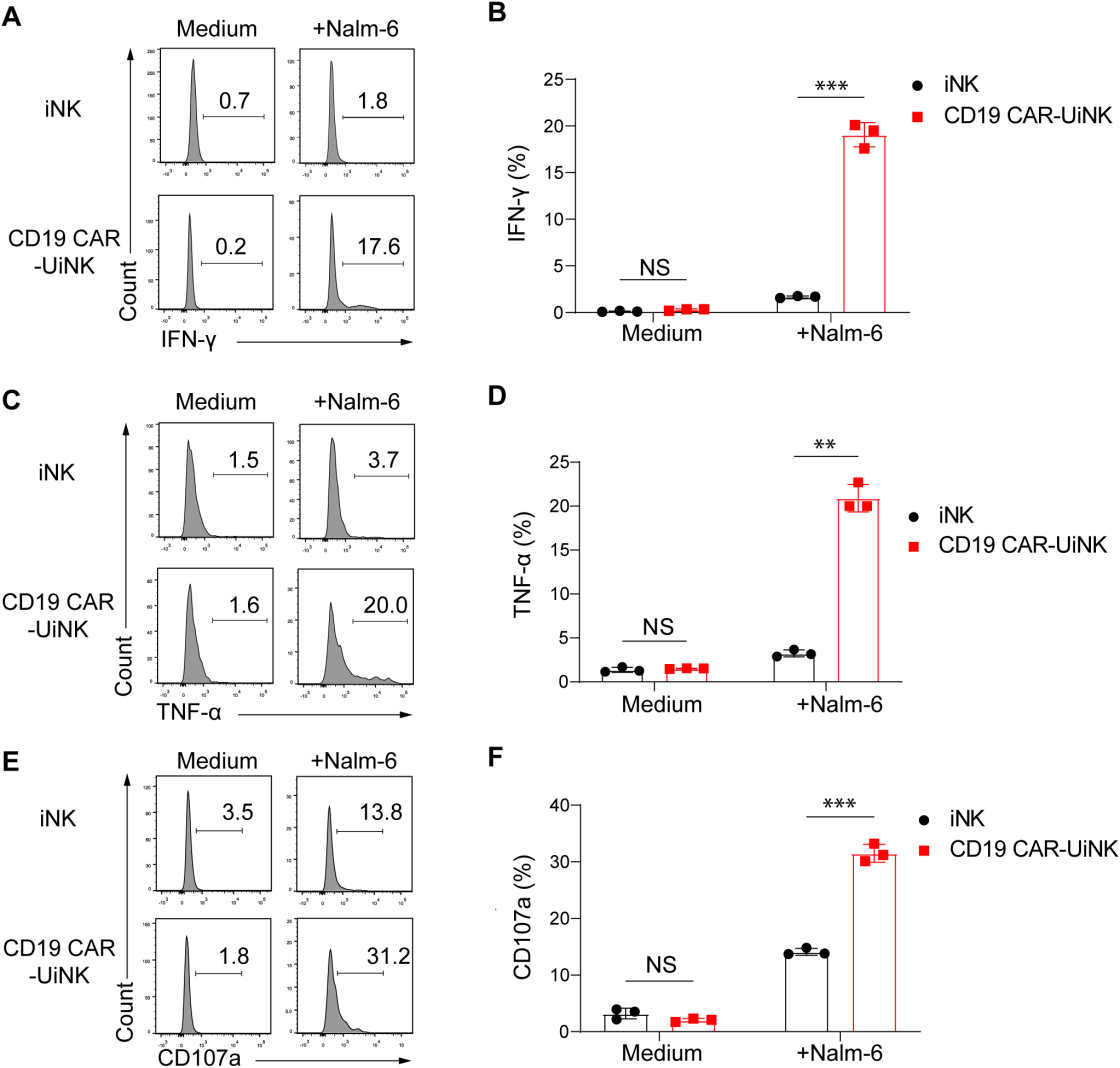
The expression of IFN-γ, TNF-α, and CD107a by iNK and CD19 CAR-UiNK cells upon stimulation with Nalm-6 tumor cells. **(A, B)** Measurement of IFN-γ production by iNK or CD19 CAR-UiNK cells in response to Nalm-6 tumor cells. The iNK or CD19 CAR-UiNK cells were stimulated by Nalm-6 at the ratio of 0.5:1 for 4 hours. Data are represented as means ± SD (n=3). NS, not significant, ***P < 0.001 (Two-tailed independent *t*-test). **(C, D)** Evaluation of TNF-α production by iNK or CD19 CAR-UiNK cells in response to Nalm-6 tumor cells. The iNK or CD19 CAR-UiNK cells were stimulated by Nalm-6 at the ratio of 0.5:1 for 4 hours. Data are represented as means ± SD (n=3). NS, not significant, **P < 0.01 (Two-tailed independent *t*-test). **(E, F)** Assessment of CD107a expression by iNK or CD19 CAR-UiNK cells following 4 hours of co-culture with Nalm-6 tumor cells at the ratio of 0.5:1. Data are represented as means ± SD (n=3). NS, not significant, ***P < 0.001 (Two-tailed independent *t*-test).

We performed the NK cell stimulation assay by co-culturing the CD19 CAR-UiNK cells with Nalm-6 (CD19^+^) tumor cells at the E: T ratio of 0.5:1 for 4 hours. The expression of IFN-γ and TNF-α, NK cell cytotoxicity-related cytokines, and CD107a, a membrane protein associated with NK cell cytotoxic activity, was analyzed after incubation. As expected, CD19 CAR-UiNK cells exhibited elevated production of IFN-γ and TNF-α compared to iNK cells when stimulated with Nalm-6 tumor cells (**Figures 4A–D**). Meanwhile, the expression of CD107a in CD19 CAR-UiNK cells was significantly higher than that observed in iNK cells when incubated with Nalm-6 tumor cells, indicating that CD19 CAR-UiNK cells released more cytotoxic granules (**Figures 4E, F**). Collectively, the CD19 CAR-UiNK cells exhibit a significant enhancement in the secretion of IFN-γ and TNF-α, as well as an increased expression of CD107a upon stimulation by tumor cells.

### 3.5 CD19 CAR-UiNK cells show robust tumor-killing abilities *in vitro*

**Figure 5:**
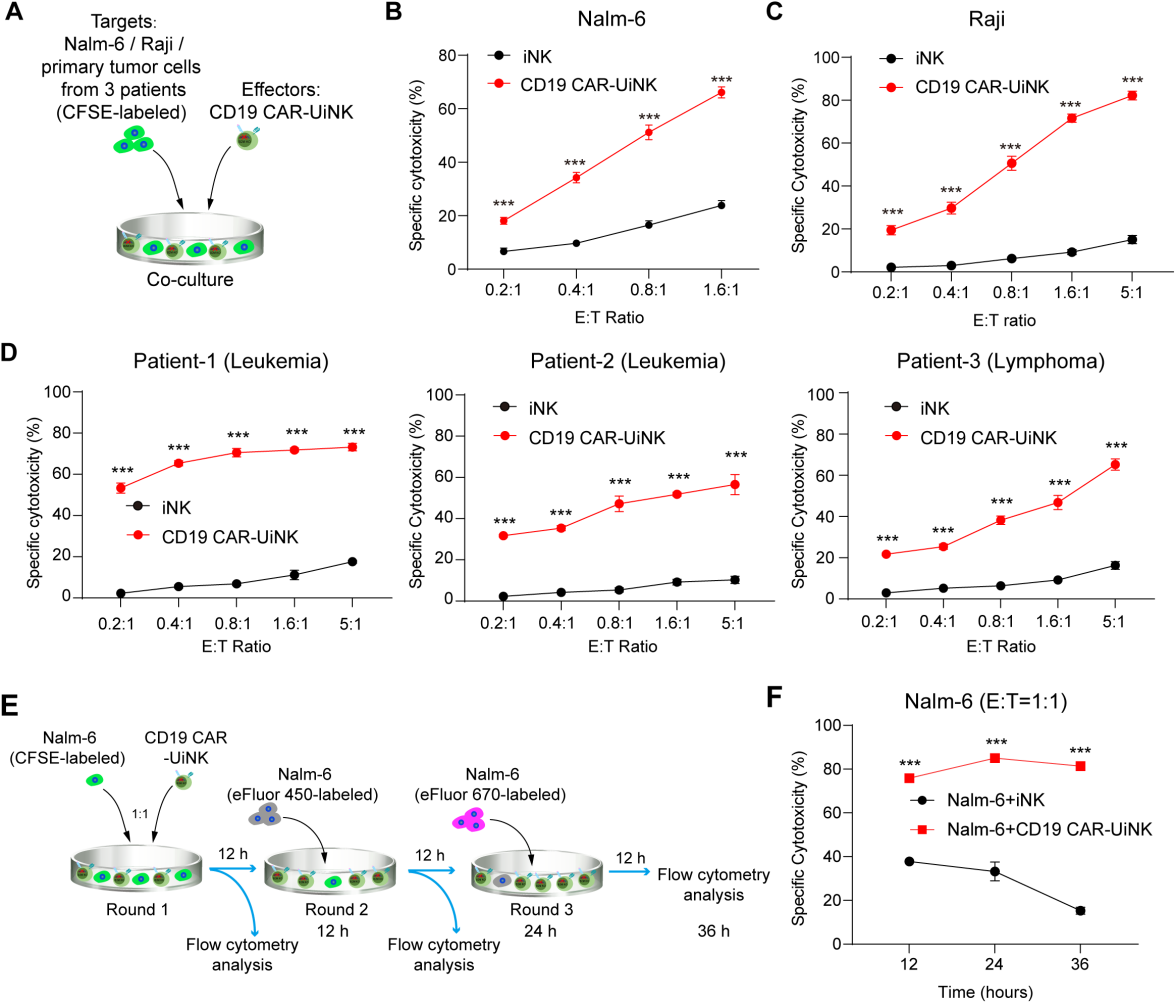
The cytotoxicity of CD19 CAR-UiNK cells against tumor cells *in vitro*. **(A)** Experimental design for the tumor-killing assay. The iNK/CD19 CAR-UiNK cells were cocultured with target cells (Nalm-6/Raji/primary tumor cells from 3 patients) labeled with CFSE. **(B)** Cytotoxicity analysis of iNK or CD19 CAR-UiNK cells against Nalm-6 tumor cells at the indicated E: T ratios after 4-hour incubation. Data are represented as means ± SD (n=3). ***P < 0.001 (Two-tailed independent *t*-test). **(C)** Cytotoxicity analysis of iNK or CD19 CAR-UiNK cells against Raji tumor cells at the indicated E: T ratios after 6-hour incubation. Data are represented as means ± SD (n=3). ***P < 0.001 (Two-tailed independent *t*-test). **(D)** Cytotoxicity analysis of iNK or CD19 CAR-UiNK cells against primary CD19^+^ leukemia or lymphoma cancer cells isolated from three patients at the indicated E:T ratios after 12-hour incubation. Data are represented as means ± SD (n=3). ***P < 0.001 (Two-tailed independent *t*-test). **(E)** Experimental design for the multiple rounds of tumor killing. The iNK/CD19 CAR-UiNK cells (effectors, E) were respectively co-cultured with Nalm-6 (targets, T) for 12 hours per round at the E: T ratio of 1:1. Fresh Nalm-6 cells were added to the iNK/CD19 CAR-UiNK cell residues incubated every other 12 hours. The Nalm-6 cells were labeled with different cell proliferation dyes in each round of incubation. **(F)** Cytotoxicity analysis of serial killing by iNK/CD19 CAR-UiNK cells. Data are represented as means ± SD (n=3). ***P < 0.001 (Two-tailed independent *t*-test).

We subsequently performed tumor-killing assays to validate the cytotoxicity of CD19 CAR-UiNK cells against tumor targets. The CD19-expressing tumor cells, including Nalm-6 (acute lymphoblastic leukemia tumor cell line), Raji (Burkitt’s lymphoma tumor cell line), and primary tumor cells from CD19-positive human B cell lymphoma and leukemia, were selected as targets for cytotoxicity assay. Tumor cells (targets, T) were co-cultured with either CD19 CAR-UiNK cells or iNK control cells (effectors, E) at various E: T ratios. (**Figure 5A**). As expected, CD19 CAR-UiNK cells were able to target Nalm-6 cells efficiently and lead to immediate apoptosis of tumor cells within 4 hours (**Figure 5B**). Meanwhile, CD19 CAR-UiNK cells showed higher cytotoxic effects than unmodified iNK cells after 6-hour co-culture with Raji (**Figure 5C**). Additionally, primary tumor cells from three patients were incubated with CD19 CAR-UiNK cells or iNK cells for 12 hours. The data revealed that CD19 CAR-UiNK cells effectively eliminated CD19^+^ primary tumor cells (**Figure 5D**). To further evaluate the sustained cytotoxicity of CD19 CAR-UiNK cells, fresh Nalm-6 tumor cells were incubated with CD19 CAR-UiNK cells or iNK cells for 12 hours at an initial ratio of 1:1 (Round 1). Subsequently, fresh Nalm-6 cells were added to the co-culture wells containing residual CD19 CAR-UiNK cells or iNK cells for another 12 hours (Round 2). The final round (Round 3) killing assay was performed as Round 2. The proportion of dead cells in targets was detected at 12, 24, and 36 hours (**Figure 5E**). Throughout these three rounds of tumor-killing assays, CD19 CAR-UiNK cells exhibited robust serial tumor-killing activity superior to iNK cells (**Figure 5F**). Taken together, CD19 CAR-UiNK cells show significantly enhanced cytotoxic activity against CD19-positive tumor cells.

### 3.6 CD19 CAR-UiNK cells suppress the tumor progress in xenograft animals

**Figure 6:**
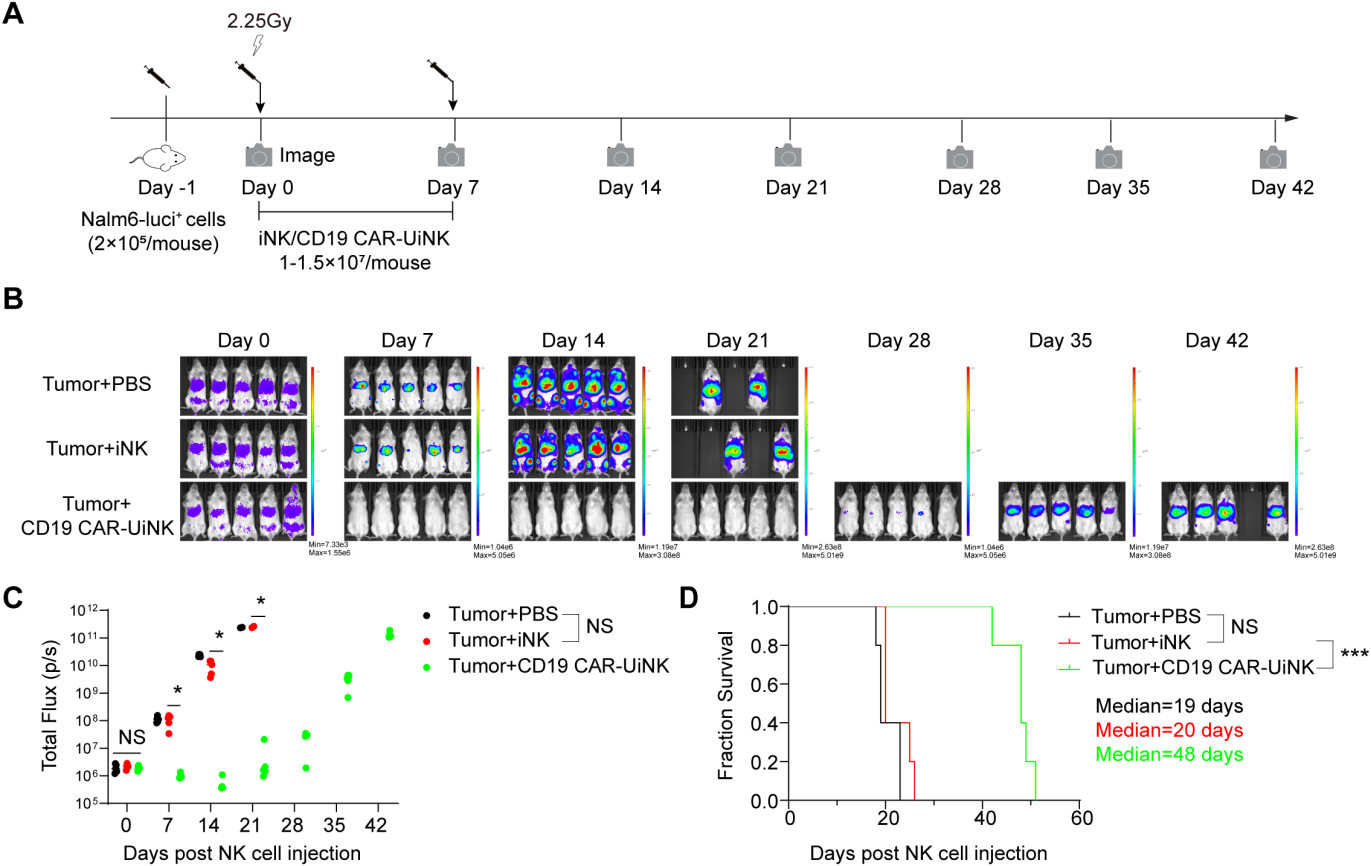
Suppression of human B leukemia progress in xenograft models by CD19 CAR-UiNK cells. **(A)** Schematic diagram of *in vivo* studies with luciferase-expressing (luci^+^) Nalm-6 cells in mouse xenograft models. The Nalm-6 tumor cells were injected into NCG mice (2×10^5^/mouse) via the tail vein on day -1. The mice were irradiated (2.25 Gy, Rad Source RS2000) on Day 0. Mice were treated intravenously with iNK cells or CD19 CAR-UiNK cells on Day 0 and Day 7. BLI was performed every week. **(B)** BLI images of the xenograft models at the indicated time points. (Tumor + PBS, Tumor + iNK, and Tumor + CD19 CAR-UiNK, n = 5 mice in each group). **(C)** Total flux (p/s) of the xenograft models measured at the indicated time points (n = 5 mice in each group). *P<0.05 (One-way ANOVA). **(D)** Kaplan-Meier survival curves of the xenograft models (n = 5 mice in each group). Median survival: Tumor + PBS, 19 days; Tumor + iNK, 20 days; Tumor + CD19 CAR-UiNK, 48 days. ***P<0.001 (Log-rank test).

To further assess the therapeutic effects of the CD19 CAR-UiNK cells on tumor cells *in vivo*, we established the B-ALL xenograft animal models by transplanting the luciferase-expressing Nalm-6 cells (Nalm-6-luci^+^) into the NCG immune-deficient mice. The NCG mice were injected with Nalm-6 tumor cells (2×10^5^/mouse) via tail vein on day -1 and received 2.25 Gy irradiation on Day 0. Subsequently, the iNK or CD19 CAR-UiNK cells were intravenously injected into the tumor-bearing animals (1-1.5 × 10^7^ iNK cells/mouse) on Day 0 and Day 7, and PBS was intravenously injected as control. Bioluminescent imaging (BLI) was performed weekly to capture the kinetics of tumor growth (**Figure 6A**). The data showed that the tumor burden of the PBS group and iNK group became increasingly severe, as indicated by the radiance and the value of total flux (**Figures 6B, C**). Eventually, both the PBS and iNK groups of mice needed ethical euthanasia due to the heavy tumor burden. However, the CD19 CAR-UiNK cells showed stronger tumor-killing ability in xenograft animals with lower radiance and total flux measurements (**Figures 6B, C**). The CD19 CAR-UiNK cell-treated mice survived significantly longer than the iNK cell-treated mice and the PBS-treated mice (Tumor + PBS: 19 days; Tumor + iNK: 20 days; Tumor + CD19 CAR-UiNK: 48 days; *P* < 0.001) (**Figure 6D**). In conclusion, these results show that the CD19 CAR-UiNK cells can efficiently suppress tumor cells and significantly prolong the survival of tumor-bearing mice.

## 4 Discussion

In this study, we tested a stable and efficient strategy for efficiently generating hypoimmunogenic CD19 CAR-UiNK cells from human ESCs. The CD19 CAR-UiNK cells overcame immune rejections by allogeneic T cells and NK cells. More importantly, the CD19 CAR-UiNK cells effectively kill B cell tumor cells and mitigate the tumor burden in xenograft animals.

The key barrier for allogeneic CAR-NK cells in the clinical setting is their limited persistence *in vivo*. Host immune rejection by T cells is considered one major determinant of the immediate clearance of allo-NK cells (50). To prolong the therapeutic effects of allo-NK and allo-CAR-NK cells, conventional approaches are to adopt lymphodepletion to suppress host immune rejection, which is a double-edged sword that increases the risk of infections and metastatic progression of certain cancers (51). To overcome host immune rejections by T cells and NK cells, we performed gene editing of the B2M locus and gene engineering at the ESC stage to further generate hypoimmunogenic CAR-NK cells. We used CRISPR/Cas12i to knock out B2M, preventing the stable expression of HLA-I molecules on the cell surface, but the HLA-encoded alpha chains are still expressed by the cell (42). It helps evade allogeneic HLA-I-mediated cytotoxicity by CD8^+^ T cells. In our study, the iNK cells generated from ESCs rarely express the HLA-II molecules, thereby avoiding rejection by CD4^+^ T cells (52). Nonetheless, the loss of HLA-I molecules in CAR-iNK cells will secondarily induce attacks by allogeneic NK cells caused by the “missing self” activation (53). Thus, we overexpressed an HLA-E single-chain trimer (HLA-E molecule fused to B2M and a peptide antigen) as an efficient approach to protect HLA-I-deficient iNK cells from NK cell lysis. This strategy has already been tested in T cells and NK cells. Shin Kaneko et al. reported the generation of hypoimmunogenic cancer-antigen-specific T cells derived from iPSCs lacking β2-microglobulin, the MHC class-II and the natural killer (NK) cell-ligand poliovirus receptor CD155, and expressing single-chain MHC class-I antigen E (19). Stefan Heinrichs et al. reported that allogeneic primary NK cells were generated by targeting B2M via a CRISPR/Cas9 lentiviral vector and equipped the HLA class I knockout NK cells with a modified single-chain HLA-E molecule (54). However, our results indicated that expressing HLA-E alone may not be sufficient to enable CD19 CAR-UiNK cells to evade attacks from allogeneic NK cells. Further expression of other HLA-I molecules, such as HLA-G/HLA-C/HLA-A, may be necessary (29, 32, 33). In addition, equipping CD19 CAR-UiNK cells with immune checkpoint (CD47 or IGSF8) may be alternative approaches to prevent allogeneic NK rejection (21, 35–38, 55). Sayandeep Saha et al. discovered that knocking out CD54 in PSC-derived cardiovascular cells can prevent the adhesion of multiple adaptive and innate effector immune cells, including T cells (56). This suggests that it is a promising strategy to protect MHC-expressing cells from T cell attacks while avoiding NK cell-mediated ‘missing-self’ killing, as MHC expression remains intact. Meanwhile, the combined deletion of adhesion ligands CD54 and CD58 in B2M^−/−^CIITA^−/−^ iPSC-derived CAR NK cells resulted in resistance to NK cell rejection, indicating that deleting adhesion ligands is a promising hypoimmune gene editing approach (57). Nevertheless, additional work is still required to explore the immune-evasion mechanisms of NK cells and resolve the bottleneck of NK cell-mediated rejection. Previous studies have demonstrated that the expression of membrane-bound IL-15/IL-15R fusion protein in NK cells significantly improved the activity and persistence of NK cells *in vivo*, ultimately enhancing their anti-tumor ability in mice (6). Deletion of CISH (a negative regulator of IL-15 signaling pathways) in iPSC-derived iNK cells can promote *in vivo* persistence and enhance the anti-tumor activity of the NK cells (58). Thus, expressing exogenous cytokines is another potential method to further improve the persistence and long-term therapeutic effect of CD19 CAR-UiNK cells.

Given the known capacity of macrophages to infiltrate and reside within solid tumors, CAR macrophages (CAR-Ms) can overcome major hurdles associated with CAR T/NK therapy, especially in solid tumors. However, CAR-Ms are still at the nascent stage with only one clinical trial initiated, and no results reported yet (59). At present, it has been reported that hPSCs can generate CAR-Ms through step-by-step induction. Macrophages are highly plastic cells that perform diverse functions in different organs. hPSC-derived CAR-Ms show strong tumor phagocytosis and killing abilities (60, 61), however, these CAR-Ms might be functionally reprogrammed by the tumor microenvironment *in vivo* to promote tumor immune evasion. Thus, CAR-Macrophages still face uncertainty in treating suitable potential tumors.

NK cells, especially primary NK cells, are difficult to modify genetically, which is an obstacle to efficiently deploying these cells as engineered immunotherapeutic agents (62). Therefore, we conducted multiple gene modifications on ESCs to obtain the hypoimmunogenic CD19 CAR-UiNK cells. The CD19 CAR-UiNK cells exhibit molecular characteristics similar to those of iNK cells and achieve comparable yields and expansion rates. It indicates that multiple gene modifications of ESCs don’t impair the differentiation potential of ESCs into NK cells. The derived CD19 CAR-UiNK cells via our strategy potentially reduce the variations in therapeutic effects caused by the heterogeneous NK cells derived from different human primary tissues. Compared with the traditional method of transducing CAR into mature NK cells (cord blood or peripheral blood-derived) with CAR-expressing viruses, this approach extremely reduces the costs of CAR engineering. Therefore, CD19 CAR-UiNK cells show promising evidence as a universal, off-the-shelf cellular therapy product for the clinical treatment of B cell leukemia or lymphoma.

This study still has some issues that need to be further explored. Currently, we have only tested the concepts of CD19 CAR-UiNK evading host immune system based on *in vitro* assays. The Hu-PBL (humanized-peripheral blood mononuclear cells) mice should be used as the recipients for *in vivo* testing of the CD19 CAR-UiNK evading human host T cell rejection (21, 57, 63). We will construct a Hu-PBL mouse model using IL-15 transgenic immunodeficient mice and transplant hypoimmunogenic cells into the mice to test our goal in the future. Through this experiment, we can better evaluate the immune compatibility of CD19 CAR-UiNK cells constructed by this strategy. The CD19 CAR-UiNK cells exhibited better cytotoxicity *in vivo* than iNK due to the ectopic expression of the CAR. Once infused *in vivo*, CAR-NK cells recognize tumor cells efficiently and kill tumor cells by releasing substances like IFN-γ and TNF-α and directly inhibiting tumor cell proliferation or inducing apoptosis (64). Previous studies reported that the cord blood derived CAR-NK cells showed comparable contributions and persistence *in vivo* to NK cells (44, 65). We will further modify the CD19 CAR-UESC to obtain CD19 CAR-UiNK cells that ectopic production of IL-15, thereby enhancing their *in vivo* proliferation, persistence, and anti-tumor activity (6, 9, 65). In our study, B2M KO-iNK cells didn’t suffer from the fratricide caused by downregulation of MHC class I. Previous studies have reported that NK cells from β2m^-/-^ mice fail to kill β2m^-/-^ normal cells, showing that they are self-tolerant (66). Inducible down-regulation of MHC class I in conditional B2M knockout mice results in NK cell tolerance without missing-self reactivity (67). It seems that during the *in vitro* induction process of iNK cells from PSC, the loss of B2M led to the absence of HLA-educating/licensing signals, which might be the reason why B2M KO iNK cells did not fratricide since they were born without the stimulation of self-HLA typical signals.

In conclusion, we offer a strategy for deriving homogeneous and hypoimmunogenic CD19 CAR-iNK cells with robust anti-tumor effects. Our study has significant implications for developing universal and affordable CD19 CAR-NK cell therapy in treating B-cell malignancies.

## Data Availability

The datasets presented in this study can be found in online repositories. The names of the repository/repositories and accession number(s) can be found in the article/**Supplementary Material**.

## References

[1] Xu KL Cheng H. CAR-NK cell therapeutics for hematologic Malignancies: hope is on the horizon. Blood Sci. (2019) 1:156–60. doi: 10.1097/BS9.0000000000000028 PMC8974902 35402810

[2] Heipertz EL Zynda ER Stav-Noraas TE Hungler AD Boucher SE Kaur N. Current perspectives on “Off-the-shelf” Allogeneic NK and CAR-NK cell therapies. Front Immunol. (2021) 12:732135. doi: 10.3389/fimmu.2021.732135 34925314 PMC8671166

[3] Lu SJ Feng Q. CAR-NK cells from engineered pluripotent stem cells: Off-the-shelf therapeutics for all patients. Stem Cell Transl Med. (2021) 10 Suppl 2:S10–S7. doi: 10.1002/sctm.21-0135 PMC8560199 34724715

[4] Zhu H Kaufman DS. Engineered human pluripotent stem cell-derived natural killer cells: the next frontier for cancer immunotherapy. Blood Sci. (2019) 1:4–11. doi: 10.1097/BS9.0000000000000023 35402797 PMC8974906

[5] Shankar K Capitini CM Saha K. Genome engineering of induced pluripotent stem cells to manufacture natural killer cell therapies. Stem Cell Res Ther. (2020) 11:234. doi: 10.1186/s13287-020-01741-4 32546200 PMC7298853

[6] Woan KV Kim H Bjordahl R Davis ZB Gaidarova S Goulding J. Harnessing features of adaptive NK cells to generate iPSC-derived NK cells for enhanced immunotherapy. Cell Stem Cell. (2021) 28:2062–75 e5. doi: 10.1016/j.stem.2021.08.013 34525347 PMC8642276

[7] Kong D Kwon D Moon B Kim DH Kim MJ Choi J. CD19 CAR-expressing iPSC-derived NK cells effectively enhance migration and cytotoxicity into glioblastoma by targeting to the pericytes in tumor microenvironment. Biomed Pharmacother = Biomed Pharmacother. (2024) 174:116436. doi: 10.1016/j.biopha.2024.116436 38508081

[8] Li Y Hermanson DL Moriarity BS Kaufman DS. Human iPSC-derived natural killer cells engineered with chimeric antigen receptors enhance anti-tumor activity. Cell Stem Cell. (2018) 23:181–92 e5. doi: 10.1016/j.stem.2018.06.002 30082067 PMC6084450

[9] Liu E Marin D Banerjee P Macapinlac HA Thompson P Basar R. Use of CAR-transduced natural killer cells in CD19-positive lymphoid tumors. N Engl J Med. (2020) 382:545–53. doi: 10.1056/NEJMoa1910607 PMC7101242 32023374

[10] Marin D Li Y Basar R Rafei H Daher M Dou J. Safety, efficacy and determinants of response of allogeneic CD19-specific CAR-NK cells in CD19(+) B cell tumors: a phase 1/2 trial. Nat Med. (2024) 30:772–84. doi: 10.1038/s41591-023-02785-8 PMC10957466 38238616

[11] Gornalusse GG Hirata RK Funk SE Riolobos L Lopes VS Manske G. HLA-E-expressing pluripotent stem cells escape allogeneic responses and lysis by NK cells. Nat Biotechnol. (2017) 35:765–72. doi: 10.1038/nbt.3860 PMC5548598 28504668

[12] Lu P Chen J He L Ren J Chen H Rao L. Generating hypoimmunogenic human embryonic stem cells by the disruption of beta 2-microglobulin. Stem Cell Rev Rep. (2013) 9:806–13. doi: 10.1007/s12015-013-9457-0 23934228

[13] Mandal PK Ferreira LM Collins R Meissner TB Boutwell CL Friesen M. Efficient ablation of genes in human hematopoietic stem and effector cells using CRISPR/Cas9. Cell Stem Cell. (2014) 15:643–52. doi: 10.1016/j.stem.2014.10.004 PMC4269831 25517468

[14] Riolobos L Hirata RK Turtle CJ Wang PR Gornalusse GG Zavajlevski M. HLA engineering of human pluripotent stem cells. Mol Ther. (2013) 21:1232–41. doi: 10.1038/mt.2013.59 PMC3677304 23629003

[15] Simpson A Hewitt AW Fairfax KA. Universal cell donor lines: A review of the current research. Stem Cell Rep. (2023) 18:2038–46. doi: 10.1016/j.stemcr.2023.09.010 PMC10679649 37832541

[16] Wang D Quan Y Yan Q Morales JE Wetsel RA. Targeted disruption of the beta2-microglobulin gene minimizes the immunogenicity of human embryonic stem cells. Stem Cell Transl Med. (2015) 4:1234–45. doi: 10.5966/sctm.2015-0049 PMC4572902 26285657

[17] Otten LA Steimle V Bontron S Mach B. Quantitative control of MHC class II expression by the transactivator CIITA. Eur J Immunol. (1998) 28:473–8. doi: 10.1002/(SICI)1521-4141(199802)28:02<473::AID-IMMU473>3.0.CO;2-E 9521055

[18] Silacci P Mottet A Steimle V Reith W Mach B. Developmental extinction of major histocompatibility complex class II gene expression in plasmocytes is mediated by silencing of the transactivator gene CIITA. J Exp Med. (1994) 180:1329–36. doi: 10.1084/jem.180.4.1329 PMC2191691 7931066

[19] Wang B Iriguchi S Waseda M Ueda N Ueda T Xu H. Generation of hypoimmunogenic T cells from genetically engineered allogeneic human induced pluripotent stem cells. Nat Biomed Eng. (2021) 5:429–40. doi: 10.1038/s41551-021-00730-z 34002062

[20] Mattapally S Pawlik KM Fast VG Zumaquero E Lund FE Randall TD. Human leukocyte antigen class I and II knockout human induced pluripotent stem cell-derived cells: universal donor for cell therapy. J Am Heart Assoc. (2018) 7:e010239. doi: 10.1161/JAHA.118.010239 30488760 PMC6405542

[21] Deuse T Hu X Gravina A Wang D Tediashvili G De C. Hypoimmunogenic derivatives of induced pluripotent stem cells evade immune rejection in fully immunocompetent allogeneic recipients. Nat Biotechnol. (2019) 37:252–8. doi: 10.1038/s41587-019-0016-3 PMC6419516 30778232

[22] Liao NS Bix M Zijlstra M Jaenisch R Raulet D. MHC class I deficiency: susceptibility to natural killer (NK) cells and impaired NK activity. Sci (New York NY). (1991) 253:199–202. doi: 10.1126/science.1853205 1853205

[23] Bix M Liao NS Zijlstra M Loring J Jaenisch R Raulet D. Rejection of class I MHC-deficient haemopoietic cells by irradiated MHC-matched mice. Nature. (1991) 349:329–31. doi: 10.1038/349329a0 1987491

[24] Braud VM Allan DS O’Callaghan CA Söderström K D’Andrea A Ogg GS. HLA-E binds to natural killer cell receptors CD94/NKG2A, B and C. Nature. (1998) 391:795–9. doi: 10.1038/35869 9486650

[25] Navarro F Llano M Bellon T Colonna M Geraghty DE Lopez-Botet M. The ILT2(LIR1) and CD94/NKG2A NK cell receptors respectively recognize HLA-G1 and HLA-E molecules co-expressed on target cells. Eur J Immunol. (1999) 29:277–83. doi: 10.1002/(SICI)1521-4141(199901)29:01<277::AID-IMMU277>3.0.CO;2-4 9933109

[26] Carosella ED Rouas-Freiss N Tronik-Le Roux D Moreau P LeMaoult J. HLA-G: an immune checkpoint molecule. Adv Immunol. (2015) 127:33–144. doi: 10.1016/bs.ai.2015.04.001 26073983

[27] King A Allan DS Bowen M Powis SJ Joseph S Verma S. HLA-E is expressed on trophoblast and interacts with CD94/NKG2 receptors on decidual NK cells. Eur J Immunol. (2000) 30:1623–31. doi: 10.1002/1521-4141(200006)30:6<1623::AID-IMMU1623>3.0.CO;2-M 10898498

[28] Shi L Li W Liu Y Chen Z Hui Y Hao P. Generation of hypoimmunogenic human pluripotent stem cells via expression of membrane-bound and secreted beta2m-HLA-G fusion proteins. Stem Cells (Dayton Ohio). (2020) 38:1423–37. doi: 10.1002/stem.3269 32930470

[29] Han X Wang M Duan S Franco PJ Kenty JH Hedrick P. Generation of hypoimmunogenic human pluripotent stem cells. Proc Natl Acad Sci United States Am. (2019) 116:10441–6. doi: 10.1073/pnas.1902566116 PMC6535035 31040209

[30] Parham P Moffett A. Variable NK cell receptors and their MHC class I ligands in immunity, reproduction and human evolution. Nat Rev Immunol. (2013) 13:133–44. doi: 10.1038/nri3370 PMC3956658 23334245

[31] Vollmers S Lobermeyer A Korner C. The new kid on the block: HLA-C, a key regulator of natural killer cells in viral immunity. Cells. (2021) 10(11):3108. doi: 10.3390/cells10113108 34831331 PMC8620871

[32] Xu H Wang B Ono M Kagita A Fujii K Sasakawa N. Targeted Disruption of HLA Genes via CRISPR-Cas9 Generates iPSCs with Enhanced Immune Compatibility. Cell Stem Cell. (2019) 24:566–78 e7. doi: 10.1016/j.stem.2019.02.005 30853558

[33] Ji TT Niu SS Fang MH Xu LX Wang X Zou J. Genome editing HLA alleles for a pilot immunocompatible hESC line in a Chinese hESC bank for cell therapies. Cell Proliferation. (2023) 56:e13471. doi: 10.1111/cpr.13471 37199039 PMC10212709

[34] Song C Wang L Li Q Liao B Qiao W Li Q. Generation of individualized immunocompatible endothelial cells from HLA-I-matched human pluripotent stem cells. Stem Cell Res Ther. (2022) 13:48. doi: 10.1186/s13287-022-02720-7 35109922 PMC8812039

[35] Deuse T Hu X Agbor-Enoh S Jang MK Alawi M Saygi C. The SIRPalpha-CD47 immune checkpoint in NK cells. J Exp Med. (2021) 218(3):e20200839. doi: 10.1084/jem.20200839 33416832 PMC7802363

[36] Hu X Gattis C Olroyd AG Friera AM White K Young C. Human hypoimmune primary pancreatic islets avoid rejection and autoimmunity and alleviate diabetes in allogeneic humanized mice. Sci Trans Med. (2023) 15:eadg5794. doi: 10.1126/scitranslmed.adg5794 37043559

[37] Hu X White K Olroyd AG DeJesus R Dominguez AA Dowdle WE. Hypoimmune induced pluripotent stem cells survive long term in fully immunocompetent, allogeneic rhesus macaques. Nat Biotechnol. (2024) 42:413–23. doi: 10.1038/s41587-023-01784-x PMC10940156 37156915

[38] Deuse T Tediashvili G Hu X Gravina A Tamenang A Wang D. Hypoimmune induced pluripotent stem cell-derived cell therapeutics treat cardiovascular and pulmonary diseases in immunocompetent allogeneic mice. Proc Natl Acad Sci United States America. (2021) 118(28):e2022091118. doi: 10.1073/pnas.2022091118 PMC8285900 34244428

[39] Pizzato HA Alonso-Guallart P Woods J Johannesson B Connelly JP Fehniger TA. Engineering human pluripotent stem cell lines to evade xenogeneic transplantation barriers. Stem Cell Reports. (2024). 19(2):299–313. doi: 10.1016/j.stemcr.2023.12.003 PMC10874864 38215755

[40] Huang D Li J Hu F Xia C Weng Q Wang T. Lateral plate mesoderm cell-based organoid system for NK cell regeneration from human pluripotent stem cells. Cell Discov. (2022) 8:121. doi: 10.1038/s41421-022-00467-2 36344493 PMC9640545

[41] Cichocki F Bjordahl R Gaidarova S Mahmood S Abujarour R Wang H. iPSC-derived NK cells maintain high cytotoxicity and enhance in vivo tumor control in concert with T cells and anti-PD-1 therapy. Sci Trans Med. (2020) 12(568):eaaz5618. doi: 10.1126/scitranslmed.aaz5618 PMC8861807 33148626

[42] Chen Y Hu Y Wang X Luo S Yang N Chen Y. Synergistic engineering of CRISPR-Cas nucleases enables robust mammalian genome editing. Innovation (Cambridge (Mass)). (2022) 3:100264. doi: 10.1016/j.xinn.2022.100264 35693153 PMC9184807

[43] Nicholson IC Lenton KA Little DJ Decorso T Lee FT Scott AM. Construction and characterisation of a functional CD19 specific single chain Fv fragment for immunotherapy of B lineage leukaemia and lymphoma. Mol Immunol. (1997) 34:1157–65. doi: 10.1016/S0161-5890(97)00144-2 9566763

[44] Wang Y Li J Wang Z Liu Y Wang T Zhang M. Comparison of seven CD19 CAR designs in engineering NK cells for enhancing anti-tumour activity. Cell Proliferation. (2024) 57:e13683. doi: 10.1111/cpr.v57.11 38830795 PMC11533075

[45] Crew MD Cannon MJ Phanavanh B Garcia-Borges CN. An HLA-E single chain trimer inhibits human NK cell reactivity towards porcine cells. Mol Immunol. (2005) 42:1205–14. doi: 10.1016/j.molimm.2004.11.013 15829309

[46] Wu P Wei H Zhang C Zhang J Tian Z. Regulation of NK cell activation by stimulatory and inhibitory receptors in tumor escape from innate immunity. Front Biosci. (2005) 10:3132–42. doi: 10.2741/1770 15970568

[47] Huntington ND Vosshenrich CA Di Santo JP. Developmental pathways that generate natural-killer-cell diversity in mice and humans. Nat Rev Immunol. (2007) 7:703–14. doi: 10.1038/nri2154 17717540

[48] Zamai L Ahmad M Bennett IM Azzoni L Alnemri ES PeRussia B. Natural killer (NK) cell-mediated cytotoxicity: differential use of TRAIL and Fas ligand by immature and mature primary human NK cells. J Exp Med. (1998) 188:2375–80. doi: 10.1084/jem.188.12.2375 PMC2212426 9858524

[49] Ni F Sun R Fu B Wang F Guo C Tian Z. IGF-1 promotes the development and cytotoxic activity of human NK cells. Nat Commun. (2013) 4:1479. doi: 10.1038/ncomms2484 23403580 PMC3586714

[50] Berrien-Elliott MM Jacobs MT Fehniger TA. Allogeneic natural killer cell therapy. Blood. (2023) 141:856–68. doi: 10.1182/blood.2022016200 PMC10023727 36416736

[51] Vivier E Rebuffet L Narni-Mancinelli E Cornen S Igarashi RY Fantin VR. Natural killer cell therapies. Nature. (2024) 626:727–36. doi: 10.1038/s41586-023-06945-1 38383621

[52] Alvaro-Benito M Freund C. Revisiting nonclassical HLA II functions in antigen presentation: Peptide editing and its modulation. Hla. (2020) 96:415–29. doi: 10.1111/tan.14007 32767512

[53] Ruggeri L Mancusi A Capanni M Urbani E Carotti A Aloisi T. Donor natural killer cell allorecognition of missing self in haploidentical hematopoietic transplantation for acute myeloid leukemia: challenging its predictive value. Blood. (2007) 110:433–40. doi: 10.1182/blood-2006-07-038687 PMC1896125 17371948

[54] Hoerster K Uhrberg M Wiek C Horn PA Hanenberg H Heinrichs S. HLA class I knockout converts allogeneic primary NK cells into suitable effectors for “Off-the-shelf” Immunotherapy. Front Immunol. (2020) 11:586168. doi: 10.3389/fimmu.2020.586168 33584651 PMC7878547

[55] Li Y Wu X Sheng C Liu H Liu H Tang Y. IGSF8 is an innate immune checkpoint and cancer immunotherapy target. Cell. (2024) 187:2703–16 e23. doi: 10.1016/j.cell.2024.03.039 38657602

[56] Saha S Haynes WJ Del Rio NM Young EE Zhang J Seo J. Diminished immune cell adhesion in hypoimmune ICAM-1 knockout pluripotent stem cells. bioRxiv: preprint Server Biol. (2024). doi: 10.1101/2024.06.07.597791 PMC12343971 40796566

[57] Hammer Q Perica K Mbofung RM van Ooijen H Martin KE Momayyezi P. Genetic ablation of adhesion ligands mitigates rejection of allogeneic cellular immunotherapies. Cell Stem Cell. (2024). 31(9):1376–1386.e8. doi: 10.1016/j.stem.2024.06.011 PMC12718542 38981470

[58] Zhu H Blum RH Bernareggi D Ask EH Wu Z Hoel HJ. Metabolic Reprograming via Deletion of CISH in Human iPSC-Derived NK Cells Promotes *In Vivo* Persistence and Enhances Anti-tumor Activity. Cell Stem Cell. (2020) 27:224–37 e6. doi: 10.1016/j.stem.2020.05.008 32531207 PMC7415618

[59] Pan K Farrukh H Chittepu V Xu H Pan CX Zhu Z. CAR race to cancer immunotherapy: from CAR T, CAR NK to CAR macrophage therapy. J Exp Clin Cancer Res. (2022) 41:119. doi: 10.1186/s13046-022-02327-z 35361234 PMC8969382

[60] Zhang J Webster S Duffin B Bernstein MN Steill J Swanson S. Generation of anti-GD2 CAR macrophages from human pluripotent stem cells for cancer immunotherapies. Stem Cell Rep. (2023) 18:585–96. doi: 10.1016/j.stemcr.2022.12.012 PMC9968983 36638788

[61] Shen J Lyu S Xu Y Zhang S Li L Li J. Activating innate immune responses repolarizes hPSC-derived CAR macrophages to improve anti-tumor activity. Cell Stem Cell. (2024) 31:1003–19 e9. doi: 10.1016/j.stem.2024.04.012 38723634

[62] Wu X Matosevic S. Gene-edited and CAR-NK cells: Opportunities and challenges with engineering of NK cells for immunotherapy. Mol Ther Oncolytics. (2022) 27:224–38. doi: 10.1016/j.omto.2022.10.011 PMC9676278 36420307

[63] Yanagisawa M Kawachi I Scannell CA Oronce CIA Tsugawa Y. Association between county-level social capital and the burden of COVID-19 cases and deaths in the United States. Ann Epidemiol. (2021) 59:21–3. doi: 10.1016/j.annepidem.2021.04.008 PMC8061785 33895244

[64] Wang W Jiang J Wu C. CAR-NK for tumor immunotherapy: Clinical transformation and future prospects. Cancer Lett. (2020) 472:175–80. doi: 10.1016/j.canlet.2019.11.033 31790761

[65] Liu E Tong Y Dotti G Shaim H Savoldo B Mukherjee M. Cord blood NK cells engineered to express IL-15 and a CD19-targeted CAR show long-term persistence and potent antitumor activity. Leukemia. (2018) 32:520–31. doi: 10.1038/leu.2017.226 PMC6063081 28725044

[66] Hoglund P Glas R Menard C Kase A Johansson MH Franksson L. Beta2-microglobulin-deficient NK cells show increased sensitivity to MHC class I-mediated inhibition, but self tolerance does not depend upon target cell expression of H-2Kb and Db heavy chains. Eur J Immunol. (1998) 28:370–8. doi: 10.1002/(SICI)1521-4141(199801)28 9485216

[67] Bern MD Parikh BA Yang L Beckman DL Poursine-Laurent J Yokoyama WM. Inducible down-regulation of MHC class I results in natural killer cell tolerance. J Exp Med. (2019) 216:99–116. doi: 10.1084/jem.20181076 30559128 PMC6314522

