# Supplementary material for: Hypoimmunogenic CD19 CAR-NK cells derived from embryonic stem cells suppress the progression of human B-cell malignancies in xenograft animals
Source: Front Immunol. 2024 Nov 27;15:1504459. doi: 10.3389/fimmu.2024.1504459 (PMC11631852; doi:10.3389/fimmu.2024.1504459)
Supplement: Supplementary file 1 [file DataSheet1.docx]

**Supplementary Figure 1**


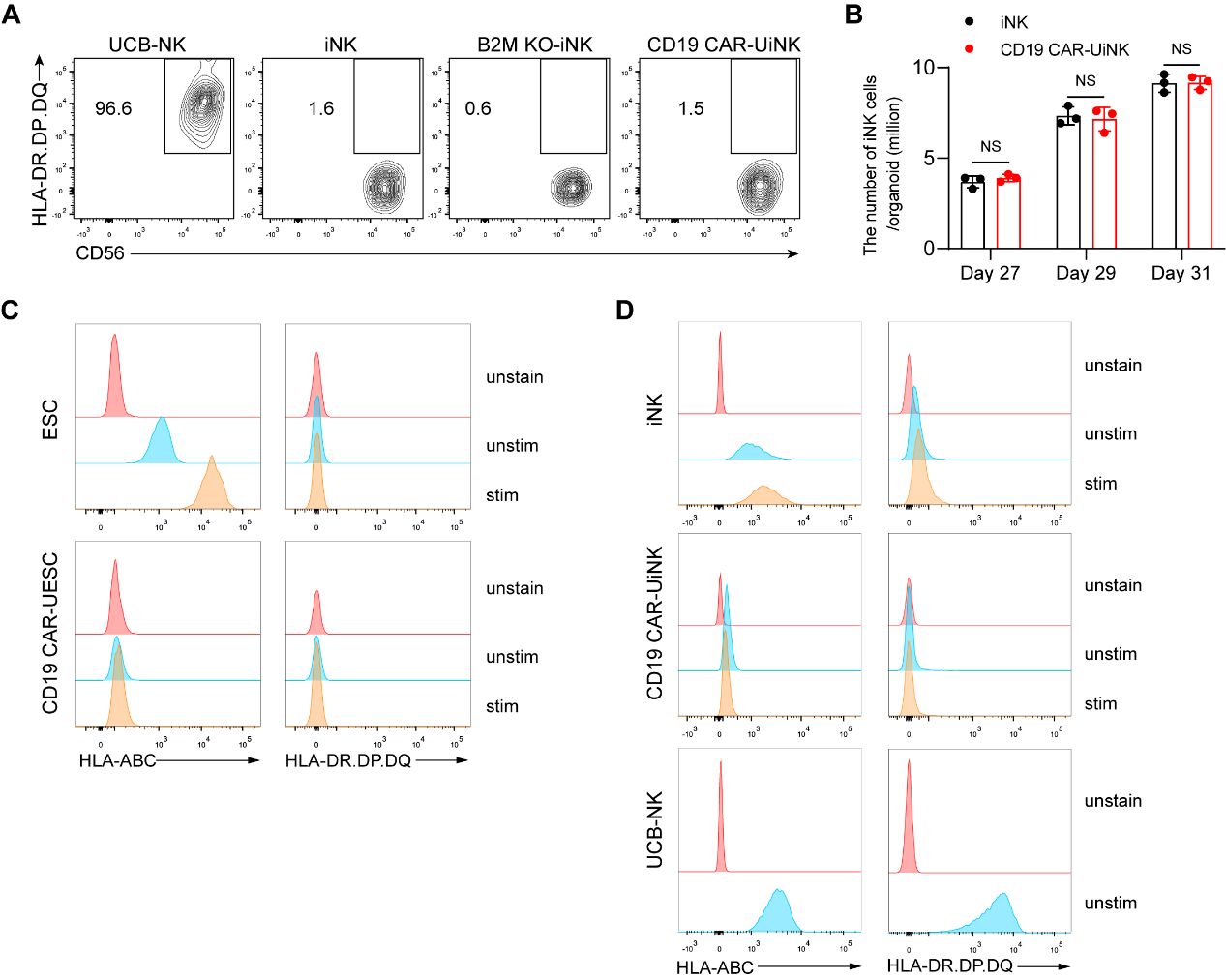


**Supplementary Figure 1. CD19 CAR-UiNK cells do not express HLA-ABC and HLA-DR.DP.DQ**

1. Flow cytometry analysis of the expression levels of HLA-DR.DP.DQ in iNK, B2M KO-iNK, or CD19 CAR-UiNK cells. UCB-NK cells were used as the positive control.
2. The dynamic quantities of iNK and CD19 CAR-UiNK cells (CD45+CD3-CD56+) at the indicated time points (n=3 organoids in each group).
3. Flow cytometry analysis of the expression levels of HLA-ABC and HLA-DR.DP.DQ in ESC and CD19 CAR-UESC cells after stimulation with IFN-γ (50ng/mL) for 48 hours.
4. Flow cytometry analysis of the expression levels of HLA-ABC and HLA-DR.DP.DQ in iNK and CD19 CAR-UiNK cells after stimulation with IFN-γ (50ng/mL) for 48 hours. The UCB-NK cells were taken as the positive control.

**Supplementary Figure 2**

**
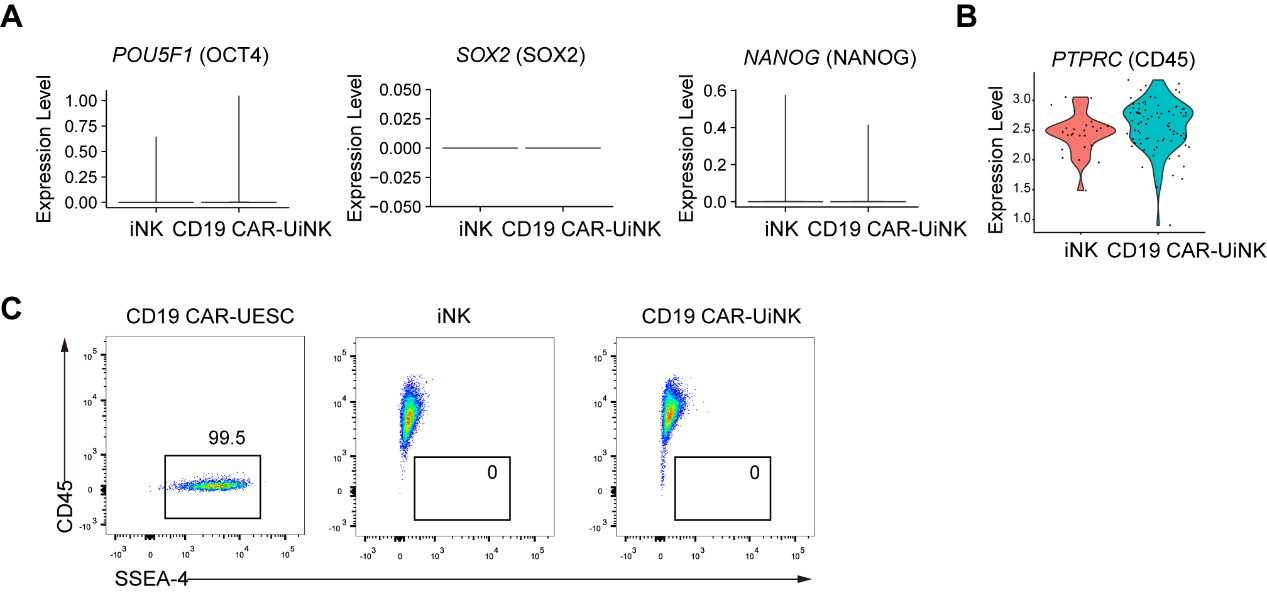
**

**Supplementary Figure 2. The expression levels of pluripotency markers in iNK and CD19 CAR-UiNK cells**

1. Violin plots showing the expression profiles of pluripotency-associated genes (*POU5F1, SOX2, NONOG*) in iNK and CD19 CAR-UiNK cells.
2. Violin plots showing the expression of profiles of *PTPRC* in the cells expressed *POU5F1* and *NANOG.*
3. Flow cytometry analysis of the expression levels of SSEA-4 in iNK and CD19 CAR-UiNK cells.

**Supplementary Figure 3**

**
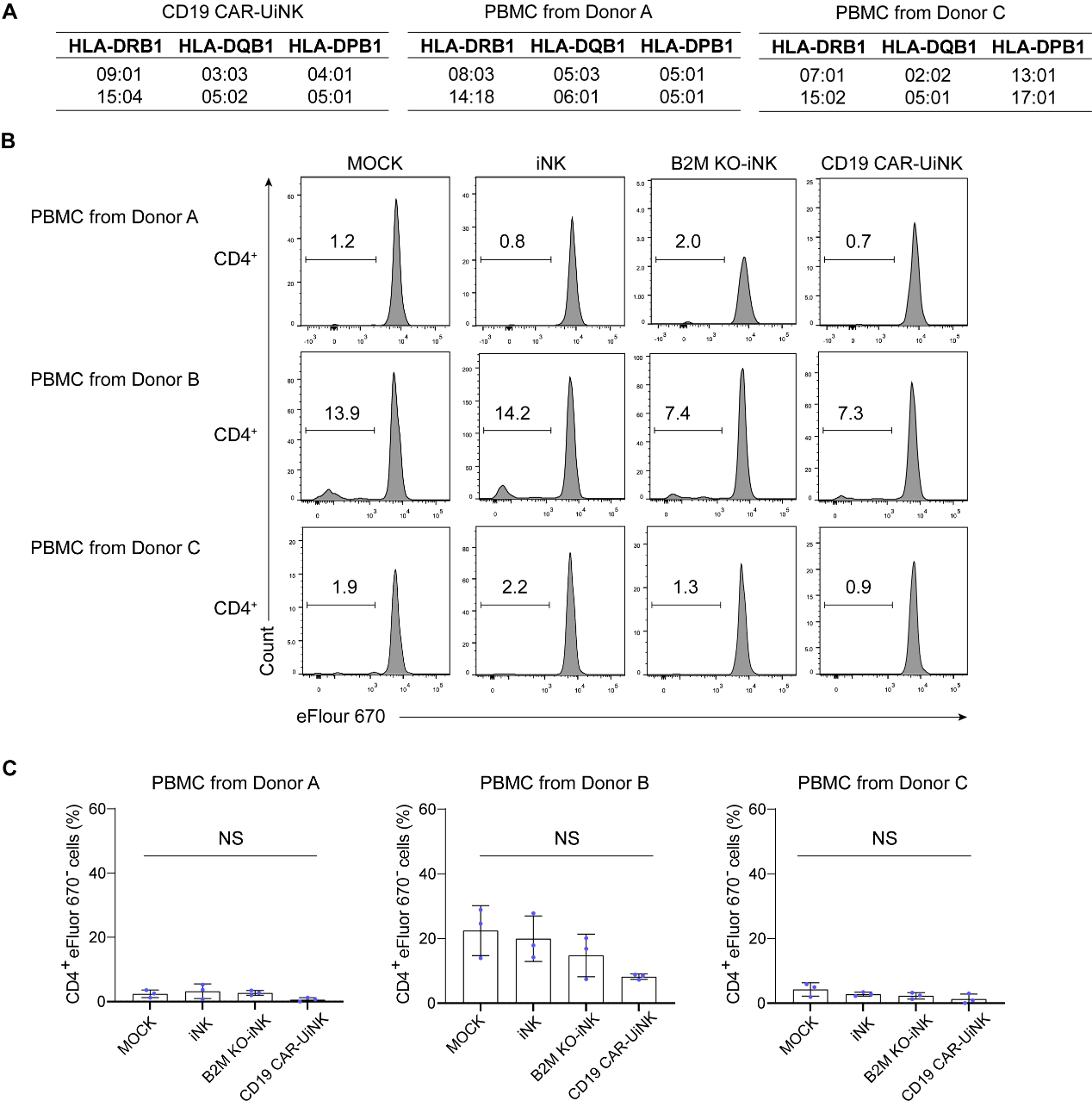
**

**Supplementary Figure 3. CD19 CAR-UiNK cells escape the response of allogeneic CD4^+^ T cells**

1. HLA genotyping table for CD19 CAR-UiNK cells and donor PBMCs. (DRB1, DQB1, and DPB1 alleles). The HLA-DRB1, DQB1, and DPB1 alleles of Donor B haven’t been detected.
2. Representative flow plots showing the percentage of proliferating CD4**^+^** T cells. PBMC from two donors (A, B, and C) were labeled with Cell Proliferation Dye eFluor 670 and co-cultured with irradiated iNK cells (iNK, B2M KO-iNK, or CD19 CAR-UiNK) for 7 days. PBMC cultured alone was defined as the MOCK group.
3. Statistical analysis of proliferating CD4**^+^** T cell (n=3 donors) populations. Data are represented as means ± SD (n=3). NS, not significant (one-way ANOVA).

**Supplementary Figure 4**

**
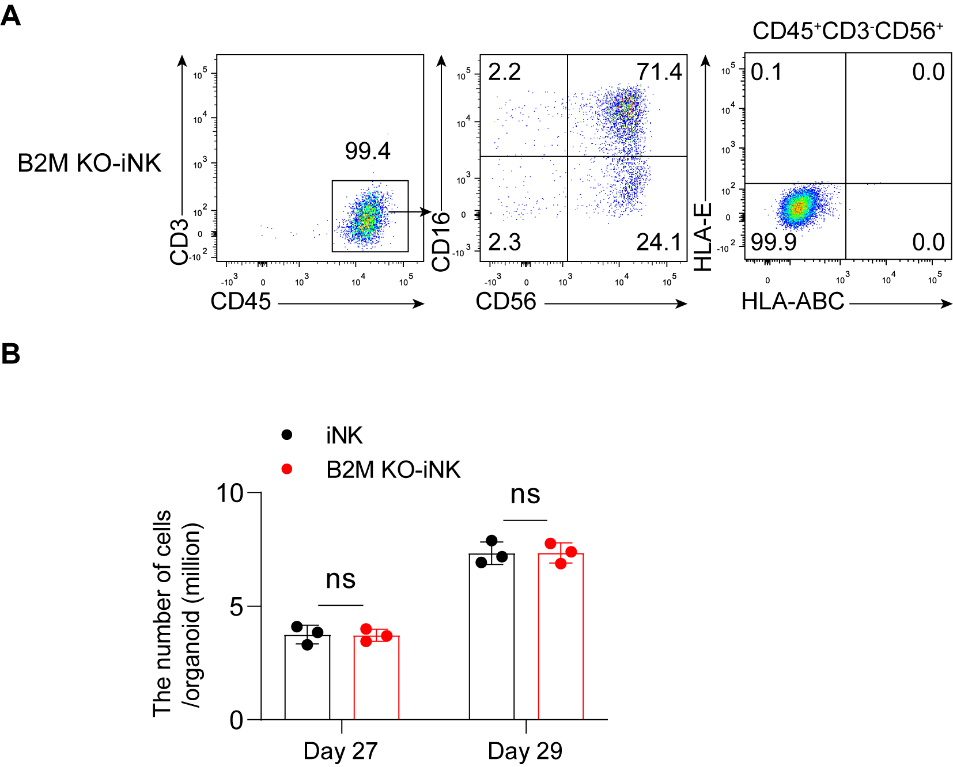
**

**Supplementary Figure 4. The phenotype and output of B2M KO-iNK cells.**

1. Flow cytometry analysis of B2M-KO iNK cells on Day 27.
2. The dynamic quantities of B2M-KO cells (CD45+CD3-CD56+) at the indicated time points (n=3 organoids in each group).
